# Difference in light use strategy in red alga between *Griffithsia pacifica* and *Porphyridium purpureum*

**DOI:** 10.1038/s41598-021-93696-6

**Published:** 2021-07-13

**Authors:** Mingyuan Xie, Wenjun Li, Hanzhi Lin, Xiaoxiao Wang, Jianwen Dong, Song Qin, Fuli Zhao

**Affiliations:** 1 School of Physics, State Key Laboratory of Optoelectronic Materials and Technologies, Sun Yat-Sen University, Guangzhou, 510275 Guangdong China; 2 Key Laboratory of Coastal Biology and Biological Resource Utilization, Yantai Institute of Coastal Zone Research, Chinese Academy of Sciences, Yantai, 264003 Shandong China; 3 Institute of Marine and Environmental Technology, University of Maryland Center for Environmental Science, Baltimore, MD 21202 USA; 4 Academy of Life Science, Qufu Normal University, Qufu, 273165 Shandong China; 5 Institute of Advanced Science Facilities, Shenzhen, 518107 Guangdong China

**Keywords:** Ultrafast photonics, Antenna complex, Biological physics

## Abstract

Phycobilisomes (PBSs) are the largest light-harvesting antenna in red algae, and feature high efficiency and rate of energy transfer even in a dim environment. To understand the influence of light on the energy transfer in PBSs, two red algae *Griffithsia pacifica* and *Porphyridium purpureum* living in different light environment were selected for this research*.* The energy transfer dynamics in PBSs of the two red algae were studied in time-resolved fluorescence spectroscopy in sub-picosecond resolution. The energy transfer pathways and the related transfer rates were uncovered by deconvolution of the fluorescence decay curve. Four time-components, i.e., 8 ps, 94 ps, 970 ps, and 2288 ps were recognized in the energy transfer in PBSs of *G. pacifica,* and 10 ps, 74 ps, 817 ps and 1292 ps in *P. purpureum.* In addition, comparison in energy transfer dynamics between the two red algae revealed that the energy transfer was clearly affected by lighting environment. The findings help us to understand the energy transfer mechanisms of red algae for adaptation to a natural low light environment.

## Introduction

Green plants, algae, and other photoautotrophs on the earth have evolved in a great variety of photosynthetic energy transfer strategies to adapt to their diverse natural habitats. These versatile strategies could occur in a broad range from the biochemical constitution of photosynthetic apparatus to those non-classical epigenetic variations on nucleotides^1^. The photosynthetic energy transfer in this functional complexity enlighten scientists to design and construct artificial systems that are able to capture, convert, and store solar energy efficiently^2,3^. However, the excited energy transfer pathways in photosynthetic apparatus remain yet poorly understood especially in the adaptation to complex environments.

Among all photosynthetic organisms, red algae live uniquely in deep-water environment where light intensity could be as low as to < 1% of that at the water surface but have a high efficiency of overall energy transfer above 95%^4^. This ability is primarily ensured by a material in light-harvesting antenna of red algae, i.e. Phycobilisomes (PBSs), with which predominant weak blue-green light in deep water could be efficiently captured and absorbed^5–7^. Biochemically, PBSs are located outside of the thylakoid membranes, and made up of water-soluble phycobiliproteins (PBPs) and linker polypeptides^8–10^. In the structure of phycobilins, PBP can be divided into four types: allophycocyanin (APC), phycocyanin (PC), phycoerythrin (PE), and phycoerythrocyanin (PEC). PBPs contain three subunits, i.e., α, β, and γ subunit (γ subunit is also known as L_R_^γ^)^11^. As PBSs and PBPs contain a large number of chromophores, energy can be transferred among chromophores theoretically. Meanwhile, the energy transfer is affected by the spatial structure of the protein, and study of energy transfer pathways will help understanding the interactions between PBPs and the linker peptides. However, because there are positive and negative energy transfer routines, the energy transfer pathway in PBSs and PBPs is very intricate.

Using spectroscopy techniques, scientists have studied the energy transfer in PBSs and energy distribution in different PBPs. Duysen et al.first revealed in steady fluorescence spectroscopy in 1951 the general excited energy transfer pathway in intact PBSs of red algae^12^. The pathway follows the sequence from peripheral PE or PEC to PC, then to APC*.* Since late 1970s, more detailed energy transfer processes have been discovered by time-resolved fluorescence spectroscopic studies^13,14^. Several ultrafast energy transfer pathways have been identified in PBSs and PBPs from red algae^15–18^. However, due to rapid energy transfer rate in PBSs and the technical bottlenecks in theoretical simulation and spectral experiment detection, the physical mechanism of photosynthetic energy transfer in PBSs has been hindered. Recently, the structures of PBS in two red macroalga *Griffithsia pacifica* (*G. pacifica*) and *Porphyridium purpureum* (*P. purpureum*), who live in different light environments, were illustrated by the cutting-edge cryo-electron microscopy (Cryo-EM) techniques at near-atom level in high resolution, providing a firm structural basis for understanding the mechanisms of energy transfer in PBSs^19,20^. With the application of ultrafast time-resolved technique, we can unveil the excited energy transfer pathways in PBSs, especially focus on the adaptation to various natural light environments.

In this study, structures of red algae *P. purpureum* and *G. pacifica*, which have been clearly understood, were selected to study the energy transfer difference within PBSs. The steady-state and time-resolved fluorescence spectra of both red algae were measured and the sequential energy flow among PBPs in PBSs was shown. The fluorescence relative decay curves of the respective transfer components were estimated by deconvolution of time-resolved spectra. The energy transfer kinetics and light adaptability of the two red algae who have different evolutionary tracks were analyzed in detail in order to explore the evolutionary adaptation of photosynthetic energy transfer within PBSs to different natural environmental habitats.

## Results and discussions

### Steady-state spectra results

**Figure 1:**
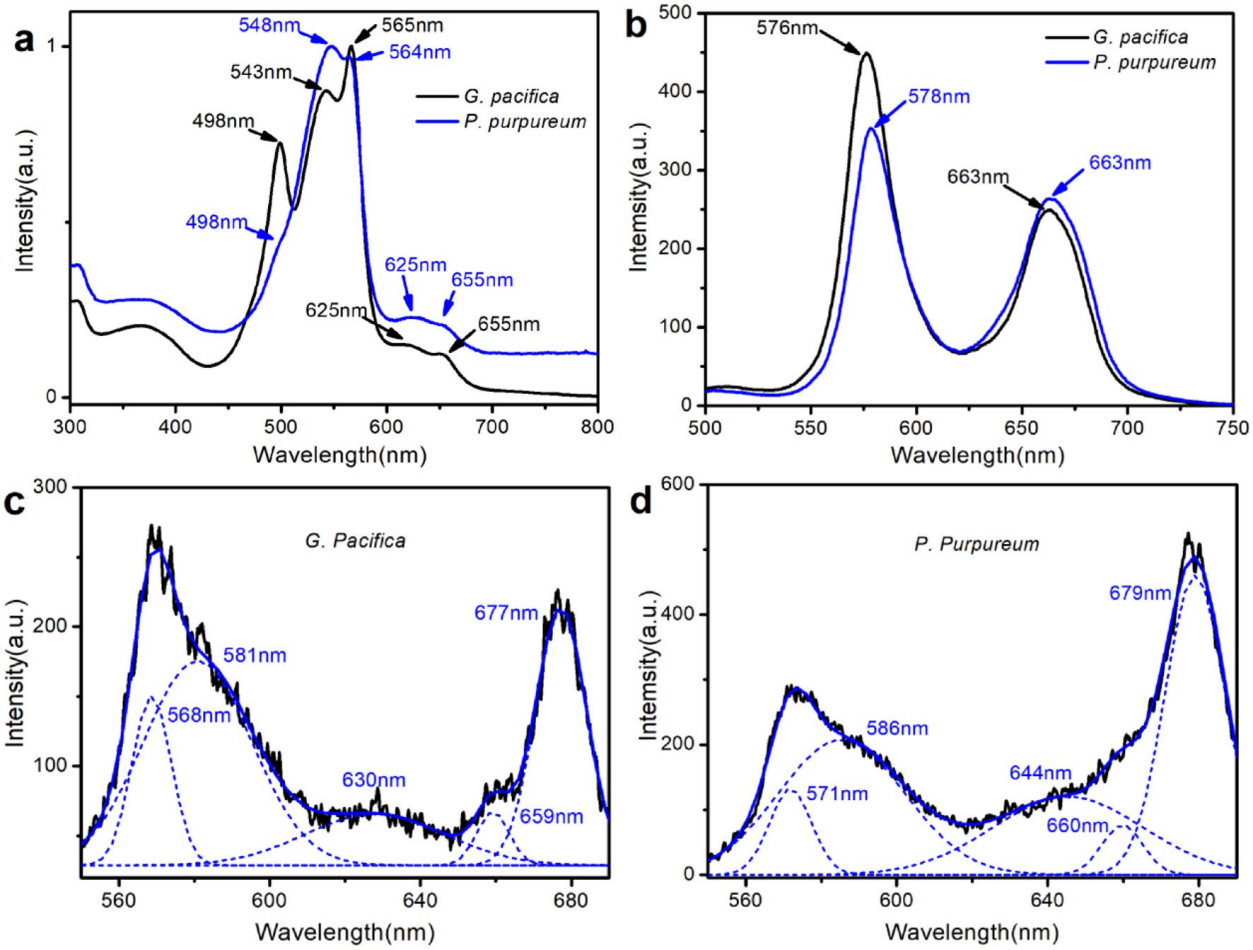
The steady-state spectra of two red algae PBSs. (**a**) The absorption spectra of *G. pacifica* PBSs and *P. purpureum* PBSs at room temperature; (**b**) The steady-state fluorescence spectra of *G. pacifica* PBSs and *P. purpureum* PBSs at room temperature*.* Excitation was done at 498 nm for both fluorescence spectra; (**c**) The fluorescence spectra of *G. pacifica* PBSs at 77 K; (**d**) The fluorescence spectra of *P. purpureum* PBSs at 77 K. Black lines represent the fluorescence data, blue dash lines represent the components well deconvoluted from fluorescence data, and blue solid lines represent the integral result of the deconvoluted components. Excitation was done at 498 nm for both fluorescence spectra.

The steady-state spectra of the two red algae PBSs were measured and analyzed in detail. Figure 1a shows the absorption spectra of PBSs from both *G. pacifica* and *P. purpureum* at room temperature. In the case of *G. pacifica* PBSs, PE exhibited three peaks at 498 nm, 543 nm, and 565 nm. These peaks reflect phycourobilin (PUB) (498 nm) and phycoerythrobilin (PEB) (543 nm and 565 nm). PC has a peak at 625 nm and APC at 655 nm, they were all originated from phycocyanobilin (PCB). While for the PBSs from *P. purpureum,* PE exhibited two peaks at 548 nm and 564 nm due to PEB existence with a shoulder at 498 nm from PUB, indicating a slight difference from those shown in the spectra of *G. pacifica* PBSs. As we know, there are two most representative PEs in red algae: B-PE and R-PE. The maximum absorption peak of B-PE is generally at 545 nm, with a shoulder at 498 nm. In R-PE, there are two distinct absorption peaks at 498 nm and 565 nm respectively. According to the above steady-state absorption spectra, it is obviously that R-PE is more dominated in *G. pacifica* PBSs than B-PE, and so does for B-PE in *P. purpureum* PBSs. Furthermore, the α subunit of B-PE contains two PEBs, the β subunit of B-PE contains three PEBs, whereas γ subunit CONTAINS two PEBs and two PUBs^21^. The type and position of chromophore in R-PE are similar to those in B-PE. The difference is that one PEB of the β subunit in R-PE is replaced by PUB. In addition, there are two types of γ subunit in R-PE. One contains a PEB and three PUBs, the other contains two PEBs and two PUBs. In red algae, the former type of γ subunit is dominant and therefore R-PE contains more PUBs, exhibiting a distinct absorption peak at 498 nm. As a result, we can see a distinct absorption peak at 498 nm in *G. pacifica* PBSs. As shown in the steady-state absorption spectra, these two type PBSs have different PBP proportions. The proportion of PE:PC:APC in PBSs of *P. purpureum* is 85.1:7.9:7.0, while that of *G. pacifica* is 90.7:5.1:4.2. It can be seen that the PE content in PBSs of *G. pacifica* is 5.6% more than that of *P. purpureum*. Therefore, the difference on the constitution of photosynthetic proteins and pigments in PBSs could be related to the habitats where the two red algae live. *G. Pacifica* usually grows in the Pacific coastal water or on other substrates in North America in depth of up to 20 m. *P. purpureum* as the only single-celled alga in Rhodophyta, is widely distributed at the surface of seawater, freshwater, brackish water, and wet land^22^. Therefore, in general, based on the distribution of the two algae, *G. Pacifica* needs more PE to absorb light in cyan and green colors, which is common in deeper seawater environment.

The fluorescence spectra of PBSs from the two algae at room temperature upon excitation at 498 nm are shown in Fig. 1b, and their emission profiles are similar. Specifically, on the fluorescence spectra, *G. pacifica* PBSs exhibited a peak of PE at 576 nm and APC at 662 nm, while *P. purpureum* PBSs was at 578 nm and APC at 662 nm. However, the PC band around 630 nm was not recognized in both the algae. The peak ratio of PE to APC was 1.82 in *G. pacifica* PBSs, while it was 1.34 in *P. purpureum* PBSs, indicating that the proportion of PE in *G. pacifica* was more than that in *P. purpureum*. This result is consistent with the structure difference of the two PBSs, whereas *G. pacifica* PBSs has more PE in the rod*.*

The fluorescence spectra of *G. Pacifica* PBSs and *P. purpureum* PBSs was measured at 77 K with excitation at 498 nm, and the results are shown in Fig. 1c, d. The fluorescence spectra of *G. pacifica* were well deconvoluted into five components at 569, 581, 630, 659, and 677 nm. The bands at 569 nm and 581 nm are from PE emission, the band at 630 nm is from PC emission, the bands at 659 nm and 677 nm are from APC and the terminal emitter L_CM_^23–25^. For *P. purpureum*, the fluorescence spectra can be well deconvoluted into five components at 571, 586, 644, 660, and 679 nm. The bands at 571 nm and 586 nm are from PE emission, the band at 644 nm is from PC emission, and the bands at 660 nm and 679 nm are from APC and terminal emitter L_CM_. The results of fluorescence deconvolution show that PE and APC of the two red algae have similar emission profiles at 77 K, but different in PC emission profiles. Furthermore, PC and APC show weaker emission compared with PE and the end emitter of the PBSs, indicating that the excitation transfer efficiency is relatively higher in PC and APC.

### Time-resolved spectra results

Fluorescence spectra in steady state are a combination of emissions from fluorescence components. Energy transfer among them shall be described on the base of their kinetics. Thus, the time-resolved fluorescence spectra of PBSs from the two red algae were analyzed in detail. The time-resolved fluorescence spectrum was conducted at 77 K, at which the molecular vibration effect and solvent effect that affect the precise reflection of energy transfer process could be neglected, allowing large signal-to-noise ratios to guarantee that the time-resolved fluorescence spectrum is close to the necessary assumption of the energy transfer in theoretical models^26^.

**Figure 2:**
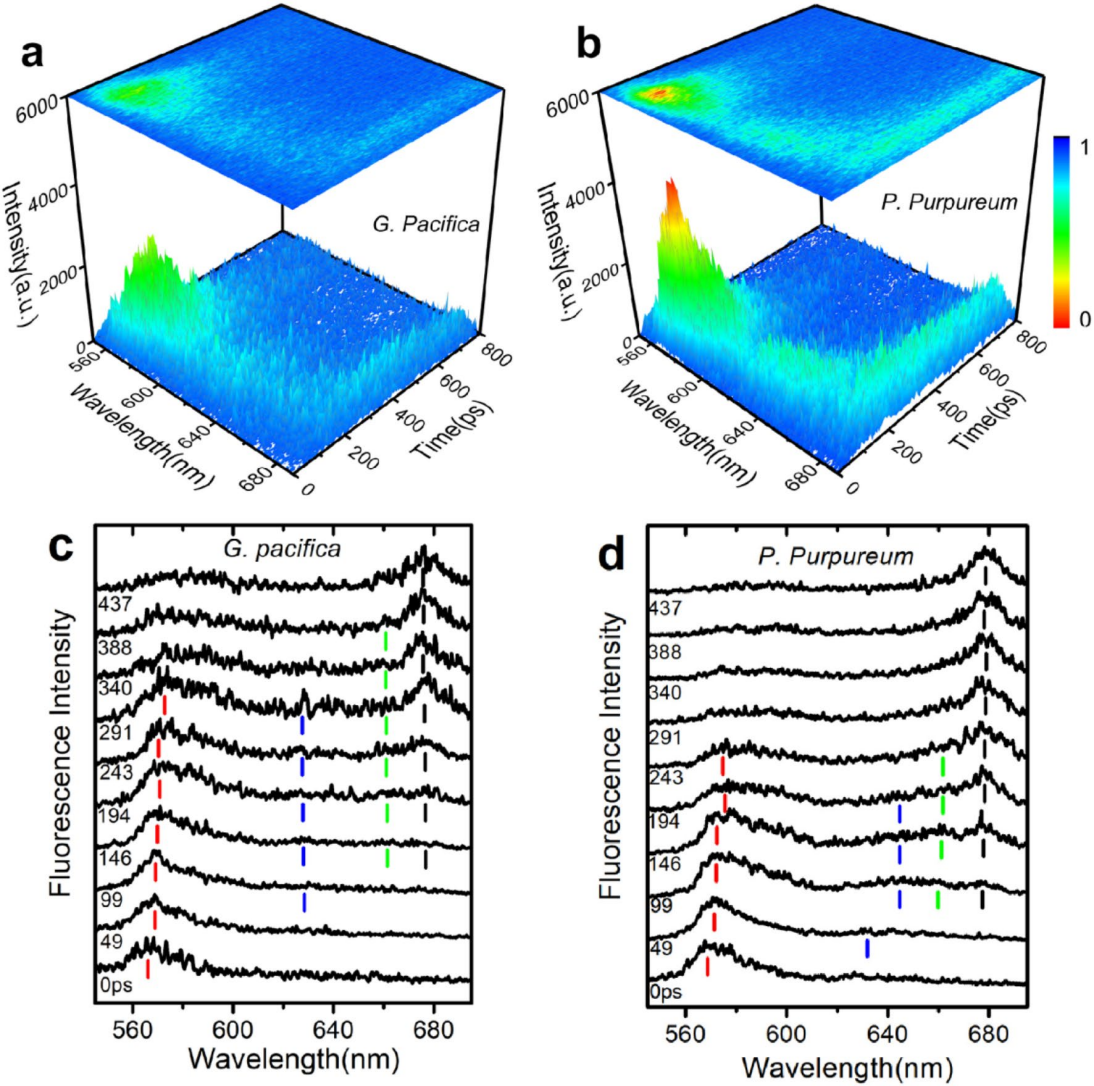
Time-resolved fluorescence spectra of two red algae PBSs. (**a**) Time-resolved fluorescence spectra of *G. pacifica* PBSs at 77 K; (**b**) Time-resolved fluorescence spectra of *P. purpureum* PBSs. Excitation was done at 498 nm.Fluorescence intensity is expressed as a gradation shown in the right side of the figure, and blue indicates a low intensity while red indicates a high intensity; (**c**) Normalized time-resolved fluorescence spectra of *G. pacifica* PBSs at 77 K; (**d**) Normalized time-resolved fluorescence spectra of *P. purpureum* PBSs at 77 K. Each spectrum is shown after normalization to the maximum intensity. Numbers in the figure show the time in picoseconds after excitation.

Figure 2a-b shows the reconstructed three-dimensional time-resolved fluorescence spectra of *G. pacifica* PBSs and *P. purpureum* PBSs at 77 K, represented in color gradation. The expression helps us to understand the energy flow conceptually. The typical feature in the spectral change of both PBSs is the relative intensity of fluorescence components. On excitation at 498 nm that preferentially excited γ subunits in PUB, the time-resolved fluorescence spectra of PBSs showed initially a spike of PE fluorescence at approximately 570 nm. A weak PC emission was then detected around 630 nm, followed by the emission from APC at 660 nm, and then by the terminal emitter at 677 nm that rose concomitantly with the decay of PE emission. Changes in the relative intensities of fluorescence components directly show the energy transfer from PE → PC → APC in both *G. pacifica* and *P. purpureum* PBSs*.* The energy transfer within PE, PC, or APC could be recognized clearly in our results.

Changes in fluorescence spectrum were much more clearly observed after normalization as shown in Fig. 2c, d. The vertical reference lines indicate the locations of the maxima. The time-resolved fluorescence was detected in synchroscan technology and the 570 nm emission was clearly detected at first in frequency domain in both spectra of *G. pacifica* and *P. purpureum* PBSs. The maximum of the fluorescence evolved the red-shift gradually. According to the research results of PBPs^21^, when PUB of PE is excited under pump wavelength of 498 nm, the excited energy could be transferred through intra-protein pathway from PUB to PEB and display a terminal emission at around 570 nm, and this intra-protein energy transfer can be very fast. The peak is then slowly red-shifted from 570 to 580 nm, and the fluorescence band broadens until it is separated into two bands. This shift pattern indicates the presence of plural components in the wavelength region. The energy transfer via preferential inter-protein pathways from PE to PC generated a terminal emission peak at 630 nm. Having been determined by Cryo-EM, *G. pacifica* PBSs has a block-shaped structure in an approximate dimension of 610 Å (length), 390 Å (height), and 380 Å (thickness)^19^, which can be distinguished from hemiellipsoidal-shaped PBSs of *P. purpureum* in dimension of approximately 680 Å (length), 390 Å (height), and 450 Å (thickness)^20^. There are more PE hexamers in *G. pacifica* PBSs than those in *P. purpureum* PBSs. The structural difference between the PBSs of the two red algae can lead to a different internal energy transfer time.

Figure 2c, d shows that the PC emission of *P. purpureum* PBSs appears at 49 ps after the excitation, approximately 50 ps earlier than that of *G. pacifica* PBSs, which indicates that the energy transfer from PE to PC in *P. purpureum* PBSs is faster than that in *G. pacifica* PBSs due to different structures of PBSs in the two red algae: fewer PE hexamers in the rods of *P. purpureum* than those in *G. pacifica*^20^. Thus, the light absorbed by PUB in PE is transported to PC over a shorter path and thus exhibits a faster energy transfer. The emission from APC and PC was detected simultaneously in both *G. pacifica* PBSs and *P. purpureum* PBSs. As we have known that in *G. pacifica* PBSs and *P. purpureum* PBSs, the rods can be divided into two types in the PBP composition. The first type consists of three PE hexamers in the part of the rod distal to the core and one PC proximal to the core, and the energy can be transported from PE to PC hexamers and to the APC in the core. The second type contains only PE proteins, and PE hexamers in the second type make extensive interactions with the α-subunits of the core APC via the rod-core linker proteins (L_RC_^2^/L_RC_^3^). Thus, the energy can be transferred directly from PE to APC in these rods. As the result, the energy from PE can be simultaneously transferred to PC and APC. In addition, the structural difference between PBSs of the two red algae results in different total energy transfer time in PBSs. The PE and PC emission of *P. purpureum* disappeared at 388 ps after the excitation as shown in Fig. 2d, indicating that the energy transfer from PE or PC to APC took place in 388 ps. However, the energy transfer time in *G. pacifica* PBSs was longer as the PE emission was detected in 437 ps after the excitation. Therefore, we can conclude that a bigger size and relative more complex structure could lead to a longer total energy transfer time in PBSs.

### Fluorescence decay-associated spectra (DAS)

To determine the constants of fluorescence decay in both the PBSs, deconvolution was conducted in a global optimization method. The time-resolved fluorescence decay at different detected wavelengths was resolved by multi-exponential deconvolution and Monte-Carlo method was adopted to process the experimental data, as shown in Supplementary Materials (Fig. S1). The fluorescence decay of *G*. *pacifica* PBSs was well fitted with five exponentials of 9, 67, 114, 793, and 2202 ps, and the amplitudes of each time component are listed in Supplementary Materials (Table S1). The fluorescence isotropic decay of *P. purpureum* PBSs was well fitted with five exponentials of 9, 73, 102, 614, and 1433 ps, and the amplitudes of each time component are listed in the Supplementary Materials (Table S2). All the time constant, except for the longest one of the terminal fluorescent emission, can be considered as a function of the time constant of energy transfer. The component (A%) with a negative amplitude corresponds to the uprising stage of fluorescence, indicative of an energy acceptor, and the component with a positive pre-exponential corresponds to the downturn stage of fluorescence, indicative of an energy donor.

**Figure 3:**
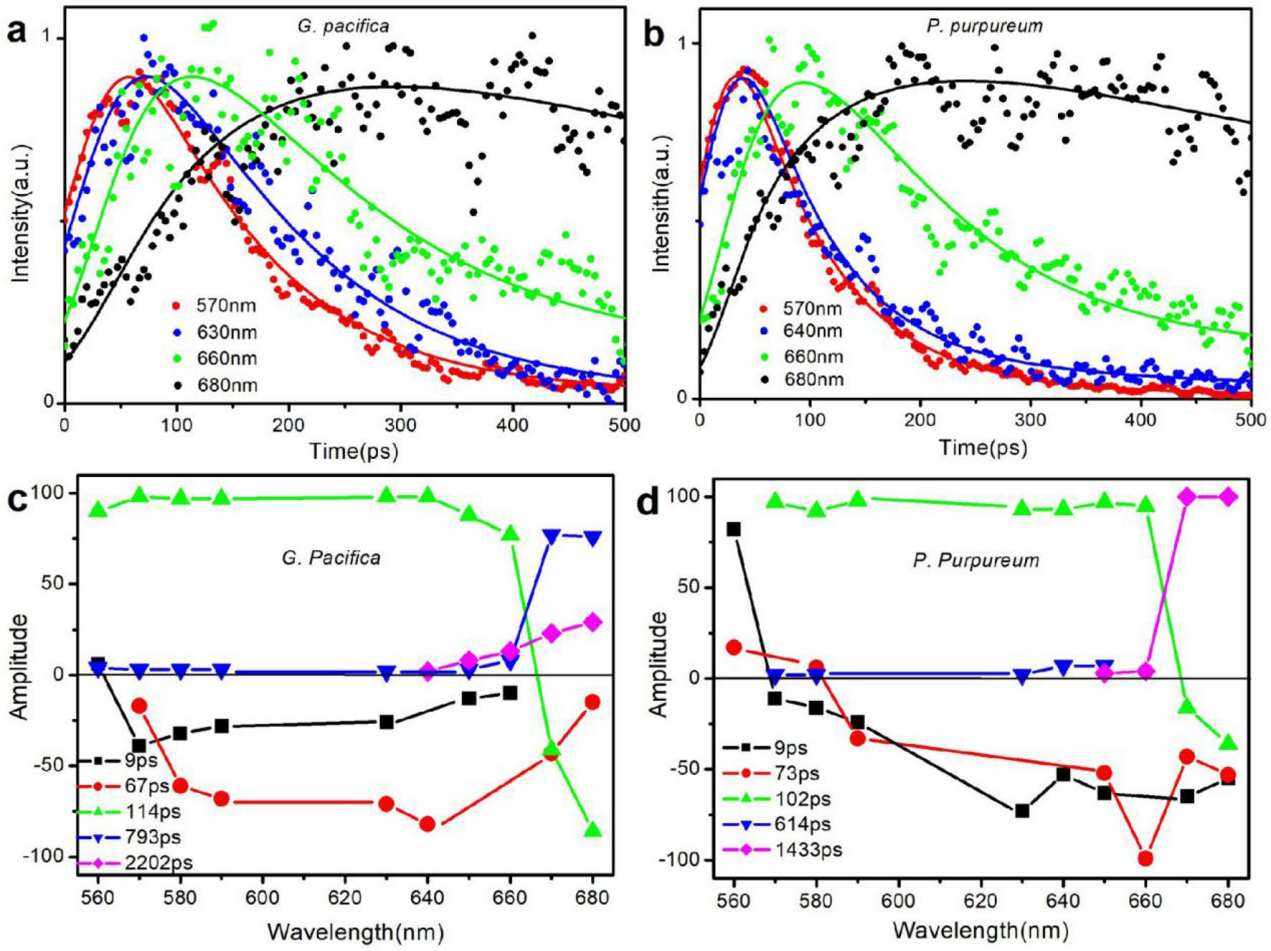
Fluorescence decay curves and DAS of two red algae PBSs. (**a**) Normalized fluorescence intensity decay curves of *G. pacifica* PBSs; (**b**) Normalized fluorescence intensity decay curves of *P. purpureum* PBSs*.* Excitation was done at 498 nm. Detection was done at 560 nm, 630 nm, 680 nm, respectively. Dots represent the experiment data, and the lines represent the fitting results; (**c**) DAS of *G. pacifica* PBSs; (**d**) DAS of *P. purpureum* PBSs*.*

Figure 3a, b shows the time-resolved fluorescence decays and the fitting results of PBSs from both *G. pacifica* and *P. purpureum* at wavelengths of 570 nm, 630 nm, 660 nm, and 680 nm. The energy transfer took place among PE, PC, APC, and the end emitter could be confirmed, because the decay at 560 nm and 630 nm corresponded to the rises at 660 nm and 680 nm from both PBSs of *G. pacifica* and *P. purpureum*. Different from the decay curves of *G. pacifica,* those of *P. purpureum* at wavelength of 570 nm and 630 nm are almost coincided, indicating a fast energy transfer between PE and PC within the *P. purpureum*.

Based on the results of the deconvolution, we obtained the fluorescence decay-associated spectra (DAS). The DAS can reveal the energy transfer dynamics among PBPs in PBSs. In DAS, the negative and positive factors indicate the kinetic increase and decrease in the populations in the excited state, respectively. The coupling of negative and positive bands was a clear indication of energy transfer from the pigments showing the positive band to those showing the negative band. Figure 3c, d shows the fluorescence decay-associated spectra from *G. pacifica* and *P. purpureum* PBSs. The results show that the two algae have similar decay constants at 10 ps, 70 ps, 100 ps, 600–800 ps, and 1 ns. According to the fluorescence decay-associated spectra results, these decay constants may be assigned as follows:

The fluorescence decay constant of 9 ps in *G. pacifica* PBSs was due to the energy transfer time in rod linker proteins L_R_^γ^, because its amplitude, shown in Fig. 3c, was positive in the blue side and then quickly turned to be negative with a minimum around 570 nm. The negative amplitude means the fluorescence rise and indicates energy transfer from donor to acceptor. The amplitude of positive signal of 9 ps was relatively smaller than that of positive signal of other lifetime component, which indicates that the energy transfer route was not the main route of energy transfer in PE. We believe that the time of 9 ps was the moment of the energy transfer from PUB to PEB in L_R_^γ^ at a fast speed in a short distance between bilins in the linker proteins. The occurrence of PEBs and PUBs in L_R_^γ^ proteins is an evolutionary adaptation to the habitats of a dark environment, because the PEBs and PUBs in L_R_^γ^ proteins increase the cross-section of antennae without expanding PBSs spacing. Different from that in *G. pacifica,* the fluorescence decay constant of 9 ps in *P. purpureum* PBSs was due to the energy transfer time between PE and PC, because its amplitude shown in Fig. 3d was positive in the blue side and quickly turned negative with a minimum around 630 nm, which is consistent with that of the *P. purpureum* PBSs in time-resolved fluorescence spectrum (Fig. 2d). As shown in the spectra, the PC emission at 630 nm could be detected at 49 ps after excitation, indicating an energy transfer pathway between PE and PC in a shorter transfer time of 49 ps. The nearly coincident fluorescence intensity decay curves at the detected wavelengths of 570 nm and 630 nm (Fig. 3b) confirmed the fast energy transfer pathway. Therefore, it is reasonable to ascribe the fast component of 9 ps to the energy transfer time between PE and PC.

For the fluorescence decay constant of 67 ps in *G. pacifica* PBSs, there was no positive signal in the wavelength region shorter than 570 nm, only a negative band was resolved with a minimum at 640 nm and a shoulder at 590 nm. The explanation is that an energy donor (D) transferred the energy to an acceptor (A1) faster than our time resolution of detection; and subsequent energy migration occurs in the same type of acceptors (D → A1 → A1). In this case, no clear positive peak of the energy donor would be observed in the shorter wavelength region of a negative band of energy acceptor (A1)^27^. Therefore, there may be an unrecognized energy donor that transferred energy faster than our time resolution of detection. PUB in PE was most probably the energy donor as shown in the steady-state absorption spectrum. Thus, as shown in the fluorescence spectra of *G*. *pacifica* PBSs, it is reasonable that the fluorescence decay constant of 67 ps could be assigned to the energy transferred from PUB to PEB chromophore and PCB chromophore. The fluorescence isotropic decay constant of 73 ps in *P. purpureum* PBSs could be assigned to the energy transfer time from PE to APC, because its amplitude shown in Fig. 3d was positive at the blue side and quickly turned negative with a minimum around 660 nm. Therefore, judging from the fluorescence spectra of the *P. purpureum* PBSs, it is reasonable to assign the decay time of 73 ps to the energy transfer time between PE and APC.

The fluorescence decay constant of 114 ps in *G. pacifica* PBSs might be assigned to the energy transfer time from rod to the terminal emitter in the core (PE to L_CM_ and PC to L_CM_)^23–25^. Shown in Fig. 3c, its amplitude, remained positive in high amplitude from 560 to 640 nm, then began to decrease at 650 nm, became negative at 670 nm, and finally ended with a minimum at 680 nm. This variation in amplitude can be described as follows: energy is transferred from a donor whose emission peak covers the range from 560 to 640 nm to an acceptor whose emission peak is at 680 nm, meaning that the energy transfer pathways from PE to L_CM_ and from PC to L_CM_ have a same transfer constant. According to the structure of G. *pacifica* PBSs^19^, type I rods (Ra/Ra’, Rb/Rb’, and Rc/Rc’) that composed of both PE and PC used the link protein L_RC_^1^ connecting with the core, whereas type II rods (Rd/Rd’, Re/Re’, Rf/Rf’, and Rg/Rg’) that composed entirely of PE used the link protein L_RC_^2^ and L_RC_^3^ connecting with the core. Thus, we believed reasonably that the energy could be transferred from both PE and PC to the core simultaneously at a same transfer rate. Furthermore, the high amplitude in both positive and negative bands indicates that the decay constant of 114 ps is the main energy transfer route that can be detected. The DAS pattern of 102 ps in *P. purpureum* PBSs shows similar features to those of 102 ps in *G. pacifica* PBSs, which might also be assigned to be the energy transfer time from rod to the end emitter in the core. However, the energy transfer pathway in *P. purpureum* PBSs exhibited a faster transfer time than that in *G. pacifica* PBSs as *P. purpureum* PBSs contained less PE hexamers in the rod and more compact structure.

As shown in the time-resolved fluorescence spectra of PBSs in *G. pacifica* (Fig. 2c), the fluorescence decay constants of 793 ps might reflect the emission of PE, because only a positive band was resolved with a maximum at 670 nm. However, the long lifetime of PE indicates that the energy transfer pathway from PE to the core is easier to saturate than that in *P. purpureum*. Because the amplitude of the decay constant of 614 ps in *P. purpureum* PBSs was < 7% in this case and it could be covered in experimental and fitted error, we did not assign the constant of 614 ps to any energy transfer process. The fluorescence isotropic decay constants of 2202 ps in *G*. *pacifica* PBSs and 1433 ps in *P. purpureum* PBSs reflect the emission of the terminal emitter L_CM_. Different from the amplitude of the constant, that of 1433 ps in *G*. *pacifica* PBSs turned out to be 100% from 670 nm, which means the L_CM_ became the only acceptor after 1433 ps, and the energy transfer in *P. purpureum* PBSs was much faster than that in the *G*. *pacifica* PBSs.

## Conclusions

**Figure 4:**
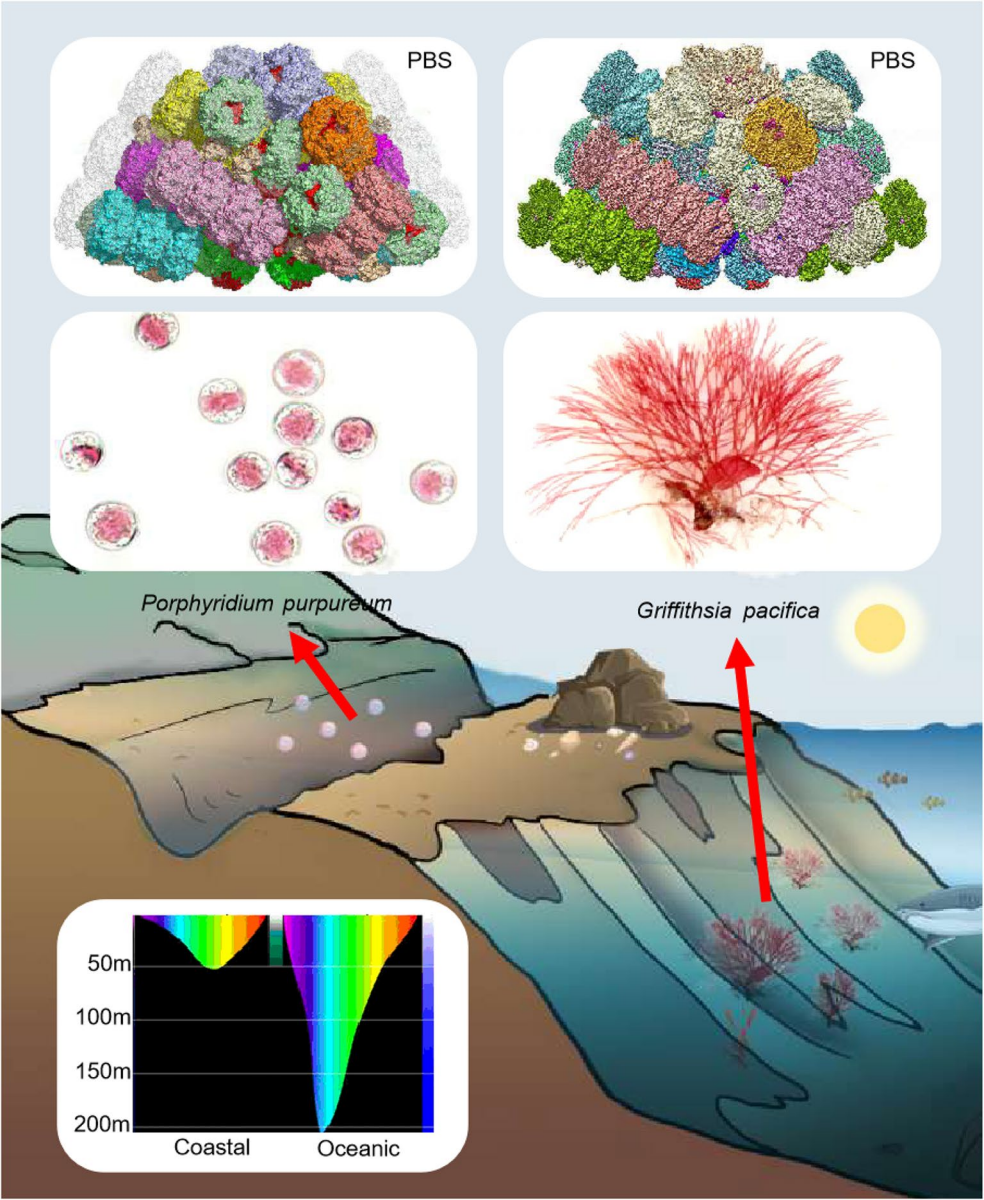
The light environment adaption of *G. pacifica* and *P. purpureum*. The top inserted 3D cryo-EM density maps of *G. pacifica* and *P. purpureum.* PBSs are reproduced from the work of Zhang et al^19^ and Ma et al^20^. The middle insert shows the microscopy images of *G. pacifica*^29^ and *P. purpureum*^30^. The bottom insert shows the light propagation characteristics in different seawater.

Among the marine macroalgae, there are more species of red algae than that of green algae or brown algae, which is closely related to the light absorption characteristics of red algae. It is known that the red and blue side of solar light was absorbed by water and phytoplankton in the top layer of the oceans, only the light in wavelength range of 400–600 nm can reach the depths up to 25 m (Fig. 4**)**. Thus, to adapt such a dim light environment, PE in absorption band at 480–580 nm becomes the main pigment in evolution, with which red algae could survive deep water. At present, red algae have been found from intertidal and subtidal to the depths up to 269 m.

Unlike *P. purpureum* who live at water surface, *G. pacifica* usually lives deep in the sea with lower light intensity and narrower light band. To adapt to such a weak light environment, *G. pacifica* have developed a block shape^28^ of PBSs with extra peripheral rods and separated hexamers composing PE in the course of evolution, which resulted in a wider range of light absorption. Such block shape with extra peripheral rods and separated hexamers results in a higher usage rate of the solar light as well as its own fluorescence light for adaption to low light intensity environment, with less chromophores fluorescence leaking during the energy transfer process. Moreover, more PE hexamers in the rod of *G. pacifica* PBS reduces the light conversion process with lower energy transfer rates from rod to the core, which helps to survive under short light duration. And more PUBs in absorption peak at 498 nm for the adaption to the light environment in mainly the wavelength of blue side. On the contrary, fewer PE hexamers and more compact structure of *P. purpureum* than those of *G. pacifica* allow faster solar light transfer from rod to core, and finally reach the reaction center. Furthermore, the energy transfer process in *G. pacifica* PBS is easier to saturation*,* which can balance the light and dark reaction for sustainable photosynthesis*.* The unique role of solar light absorption and energy transfer behavior is irreplaceable, and contributed greatly to in marine ecosystem.c

## Methods

### Growth of red algae and isolation of PBSs

PBSs were prepared according to the methods described in our previous studies^19,20^.

### Determination of steady-state and time-resolved spectral properties

Absorption and steady-state fluorescence spectra were recorded by a spectrophotometer (Persee TU-1810c) and a spectrofluorometer (Hitachi FL-4500), respectively. The spectral sensitivity of the fluorometer was corrected by the radiation profile of the standard lamp. The time-resolved fluorescence spectra (TRFS) were measured at 77 K with a synchroscan streak-camera (Hamamatsu C6860, time-resolution 700 fs) coupled with a polychromator (Supplementary Materials Fig. S2). Silica cuvettes containing samples were placed in liquid nitrogen and frozen in the dark. The light source was a Ti (sapphire laser, excitation wavelength of 498 nm) for excitation of phycobiliproteins. Spectral data were measured in a 0.2-nm interval and stored, and the time-resolved fluorescence spectra were reconstructed afterwards.

### Analysis of the time-resolved fluorescence spectrum

DAS were calculated as follows: fluorescence decay curves were deconvoluted based on a global optimization method^31^. After deconvolution, the time resolution was enhanced to approximately 2 ps. All the decays were re-analyzed using the same lifetime parameters. The amplitudes of these exponential components as a function of emission wavelength provided DAS^32^.

The fluorescence detected by the streak camera was transferred into computer for deconvolution. The detected fluorescence intensity *F*_exp_ can be described as follows:

1 $$F_{{{\text{exp}}}} \left( t \right) = f_{{{\text{pump}}}} \otimes F_{{{\text{theo}}}} = \int {f_{{{\text{pump}}}} } \left( t \right)F_{{{\text{theo}}}} \left( {t - t^{'} } \right)dt'$$

where *f*_pump_ represents the pump laser pulse. Here we considered the theoretical fluorescence intensity *F*_theo_ as a sum of multi-exponential form:

2 $$F_{{{\text{theo}}}} \left( t \right) = \sum {\varepsilon _{i} \exp \left( { - {t \mathord{\left/ {\vphantom {t {\tau _{i} }}} \right. \kern-\nulldelimiterspace} {\tau _{i} }}} \right)}$$

We applied a deconvolution procedure based on a global optimization method, which is described as follows to fit the isotropic fluorescence^31^. Letter A denotes the amplitude of the fluorescence isotropic decay constant of *τ*_*i*_*.*

3 $$I_{{\text{n}}} \left( {\lambda ,t} \right) = \sum\limits_{{i = 1}}^{n} {A_{i} \left( {\lambda _{i} } \right)\exp \left( {{{ - t} \mathord{\left/ {\vphantom {{ - t} {\tau _{i} }}} \right. \kern-\nulldelimiterspace} {\tau _{i} }}} \right)}$$

## Supplementary Information

Supplementary file.
