# Supplementary material for: Difference in light use strategy in red alga between Griffithsia pacifica and Porphyridium purpureum
Source: Sci Rep. 2021 Jul 13;11:14367. doi: 10.1038/s41598-021-93696-6 (PMC8277835; doi:10.1038/s41598-021-93696-6)
Supplement: Supplementary file 1 — Supplementary file. [file 41598_2021_93696_MOESM1_ESM.docx]

Supplementary Materials

# Difference in light use strategy in red alga between *Griffithsia pacifica* and *Porphyridium purpureum*

Mingyuan Xie^1,5,6^, Wenjun Li^2,6^, Hanzhi Lin^3,6^, Xiaoxiao Wang^2,4^,

Jianwen Dong^1^, Song Qin^2,4,7^& Fuli Zhao^1,7^

^1^School of physics, State Key Laboratory of Optoelectronic Materials and Technologies, Sun Yat-sen University, Guangzhou, 510275, Guangdong, China. ^2^Key Laboratory of Coastal Biology and Biological Resource Utilization, Yantai Institute of Coastal Zone Research, Chinese Academy of Sciences, Yantai, 264003, Shandong, China. ^3^Institute of Marine and Environmental Technology, University of Maryland Center for Environmental Science, Baltimore, 21202, Maryland, U.S. ^4^Academy of Life Science, Qufu Normal University, Qufu, 273165, Shandong, China. ^5^Institute of advanced Science Facilities, Shenzhen, 518107, Guangdong, China. ^6^These authors contributed equally: Mingyuan Xie, Wenjun Li, Hanzhi Lin. ^7^These authors jointly supervised this work: Song Qin, Fuli Zhao.

Correspondence author:

Song Qin([sqin@yic.ac.cn](mailto:sqin@yic.ac.cn)),

Fuli Zhao (stszfl@mail.sysu.edu.cn).

Supplementary Figures:


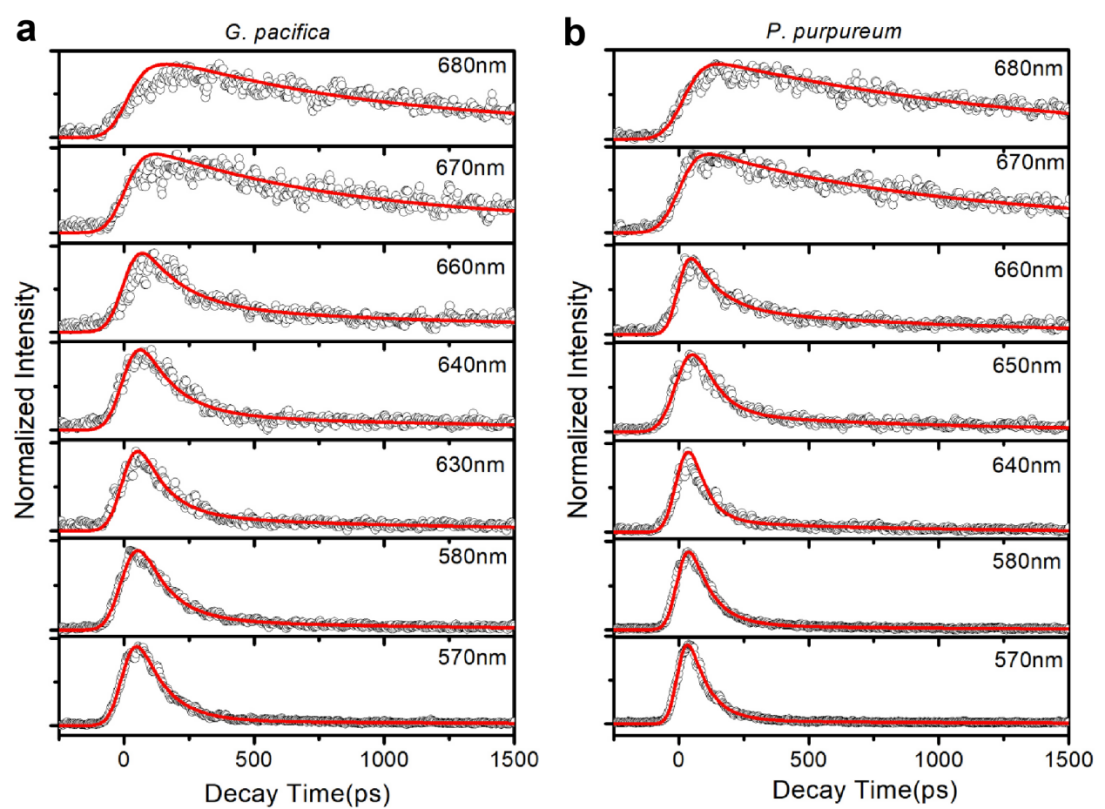


**Fig. S1.** Normalized fluorescence intensity decay curves of the two red algae PBSs.

(a) Normalized fluorescence intensity decay curves of G. pacifica PBSs. (b) Normalized fluorescence intensity decay curves of P. purpureum PBSs. Excitation was done at 498nm. Black circles represent the experiment data, and the red line represent the fitting results. Numbers in the figure show the detection wavelength.

**Fig. S2.** Experimental setup for time-resolved spectra measurement

Supplementary Tables:

**Table S1.** The deconvolution results of *G. pacifica* PBSs. Excitation was done at 498nm.

| Red Algae | E_m_/nm | τ_1_/ps | A_1_/% | τ_2_/ps | A_2_/% | τ_3_/ps | A_3_/% | τ_4_/ps | A_4_/% | Τ_5_/ps | A_5_/% |
| --- | --- | --- | --- | --- | --- | --- | --- | --- | --- | --- | --- |
| ***G. p*** | 560 | 8 | 6 | 76 | 90 | 694 | 4 | - | - | - | - |
|  | 570 | 11 | -39 | 116 | 98 | 807 | 3 | 60 | -17 | - | - |
|  | 580 | 7 | -32 | 116 | 97 | 740 | 3 | 63 | -61 | - | - |
|  | 590 | 9 | -28 | 117 | 97 | 777 | 3 | 73 | -68 | - | - |
|  | 630 | 8 | -26 | 111 | 98 | 850 | 2 | 62 | -71 | - | - |
|  | 640 | - | - | 116 | 98 | - | - | 78 | -82 | 1979 | 2 |
|  | 650 | 6 | -13 | 100 | 88 | 993 | 3 | - | - | 2560 | 8 |
|  | 660 | 11 | -10 | 154 | 77 | 947 | 8 | - | - | 1909 | 13 |
|  | 670 | - | - | 104 | -41 | 654 | 77 | 62 | -43 | 1619 | 23 |
|  | 680 | - | - | 132 | -86 | 677 | 70 | 73 | -15 | 2311 | 29 |
|  |  | 9 |  | 114 |  | 793 |  | 67 |  | 2202 |  |

**Table S2.** The deconvolution results of *P. purpureum* PBSs. Excitation was done at 498nm.

| Red Algae | E_m_/nm | τ_1_/ps | A_1_/% | τ_2_/ps | A_2_/% | τ_3_/ps | A_3_/% | τ_4_/ps | A_4_/% | Τ_5_/ps | A_5_/% |
| --- | --- | --- | --- | --- | --- | --- | --- | --- | --- | --- | --- |
| ***P. p*** | 560 | 7 | 82 | 80 | 17 | - | - | - | - | - | - |
|  | 570 | 7 | -11 | - | - | 554 | 2 | 78 | 97 | - | - |
|  | 580 | 12 | -16 | 91 | 6 | 621 | 2 | 106 | 92 | - | - |
|  | 590 | 12 | -24 | 64 | -33 | - | - | 107 | 98 | - | - |
|  | 630 | 10 | -73 | - | - | 866 | 2 | 85 | 93 | - | - |
|  | 640 | 9 | -53 | - | - | 485 | 7 | 72 | 93 | - | - |
|  | 650 | 8 | -63 | 83 | -52 | 545 | 7 | 141 | 97 | 1410 | 3 |
|  | 660 | - | - | 61 | -99 | - | - | 106 | 95 | 2065 | 4 |
|  | 670 | 9 | -65 | 58 | -43 | - | - | 109 | -16 | 1076 | 100 |
|  | 680 | 7 | -55 | 77 | -53 | - | - | 117 | -36 | 1181 | 100 |
|  |  | 9 |  | 73 |  | 614 |  | 102 |  | 1433 |  |

Supplementary for sample culture and growth

**Preparation of PBS from G. pacifica:**

To obtain the high-resolution structure, we screened PBSs from several algal species for homogeneity. The PBS from **G. pacifica** exhibited a very compact and uniform structure, as indicated by the raw micrographs of the cryo-EM PBS samples and the 2D class averages of the cryo-EM particles. Griffithsia pacifica (From UTEX Culture Collection of Algae) was cultured in Enrichment Seawater Medium(UTEX,<https://utex.org/products/enrichmentsolution->for-seawater-medium-recipe) bubbled with sterilizing filtered air at 22 °C, under a light–dark period of 16 h: 8 h, with illumination from cool-white fluorescent lamps at a light intensity of approximately 50 μ mol photons m^−2^ h^−1^. Algal tissue was suspended in Buffer A (0.65 M Na/K-PO_4_ buffer with 0.5 M sucrose and 10 mM EDTA, pH 7.0) at 0.3 g of wet weight ml^−1^. The tissue was homogenized at room temperature using a homogenizer and pestle, and was further ultrasonicated for 10 min in the presence of 1 mM phenylmethylsulfonyl fluoride (PMSF). Triton X-100 was added to the suspension at a final concentration of 2% v/v and incubated for 30–40 min at room temperature followed by centrifugation at 20,000g for 30 min at 18 °C. The middle aqueous violet solution was loaded onto a discontinuous sucrose gradient (2 ml of 0.5 M, 2 ml of 0.75 M, 2 ml of 1.0 M, 2 ml of 1.5 M, 1 ml of 2.0 M, all in Buffer B: 0.75 M K/NaPO_4_ buffer with 10 mM EDTA, pH 7.0). The samples were centrifuged at 120,000g for 4 h at 18 °C using a SW41 rotor on a Beckman Coulter Optima XPN-100 centrifuge. Two visible bands were obtained after centrifugation, with band 1 as the main layer of intact PBSs.

**Preparation of PBS from P. purpureum:**

P. purpureum (From UTEX Culture Collection of Algae, UTEX 2757) was cultured in Bold 1NV: Erdshreiber (1:1) half-seawater medium, bubbled with sterilizing filtered air at 22 °C, under a 16 h:8 h light–dark cycle, with a white-light flux of about 37 μmol photons per m^2^ per second. Algal cells were collected by centrifugation for 10 min at 6,000g, and resuspended in Buffer A (0.65 M Na/KPO_4_ buffer with 0.5 M sucrose and 10 mM EDTA, pH 7.0) at 0.3 g of wet weight per ml. Then cells were homogenized twice at 4 °C using a French Press (EmulsiFlex-C3, Avestin) at 4,000 p.s.i., and phenylmethylsulfonyl fluoride was added to a final concentration of 1 mM. After 30 min of incubation with lauryldimethylamine N-oxide (Sigma) (48 mg g^−1^ wet algal cells), debris and supernatant chlorophyll were removed by centrifugation at 20,000g for 30 min at 18 °C. The middle aqueous violet solution was loaded in a discontinuous sucrose gradient (2 ml of 0.5 M, 2 ml of 0.75 M, 2 ml of 1.0 M, 2 ml of 1.5 M, 1 ml of 2.0 M, all in Buffer B: 0.75 M K/NaPO_4_ buffer with 10 mM EDTA, pH 7.0) and spun at 120,000g for 4 h at 18 °C using a SW41 rotor on Optima XPN-100 centrifuge (Beckman Coulter). Three visible bands were obtained after centrifugation and violet band 1 is the main layer of intact PBSs.
